# Use of a real-time risk-prediction model to identify pediatric patients at risk for thromboembolic events: study protocol for the Children’s Likelihood Of Thrombosis (CLOT) trial

**DOI:** 10.1186/s13063-022-06823-7

**Published:** 2022-10-22

**Authors:** Shannon C. Walker, Benjamin French, Ryan Moore, Henry J. Domenico, Jonathan P. Wanderer, Sreenivasa Balla, C. Buddy Creech, Daniel W. Byrne, Allison P. Wheeler

**Affiliations:** 1 Division of Pediatric Hematology/Oncology, Vanderbilt University Medical Center, Nashville, USA; 2 Department of Pathology, Microbiology, and Immunology, Vanderbilt University Medical Center, Nashville, USA; 3 Department of Biostatistics, Vanderbilt University Medical Center, Nashville, USA; 4 Department of Anesthesiology and Biomedical Informatics, Vanderbilt University Medical Center, Nashville, USA; 5 HealthIT, Vanderbilt University Medical Center, Nashville, USA; 6 Vanderbilt Vaccine Research Program and Division of Pediatric Infectious Diseases, Vanderbilt University Medical Center, Nashville, USA

**Keywords:** Pediatrics, Hematology, Pragmatic trials, Study design and best practice

## Abstract

**Background:**

Pediatric patients have increasing rates of hospital-associated venous thromboembolism (HA-VTE), and while several risk-prediction models have been developed, few are designed to assess all general pediatric patients, and none has been shown to improve patient outcomes when implemented in routine clinical care.

**Methods:**

The Children’s Likelihood Of Thrombosis (CLOT) trial is an ongoing pragmatic randomized trial being conducted starting November 2, 2020, in the inpatient units at Monroe Carell Jr. Children’s Hospital at Vanderbilt in Nashville, TN, USA. All admitted patients who are 21 years of age and younger are automatically enrolled in the trial and randomly assigned to receive either the current standard-of-care anticoagulation practice or the study intervention. Patients randomized to the intervention arm are assigned an HA-VTE risk probability that is calculated from a validated VTE risk-prediction model; the model is updated daily with the most recent clinical information. Patients in the intervention arm with elevated risk (predicted probability of HA-VTE ≥ 0.025) have an additional review of their clinical course by a team of dedicated hematologists, who make recommendations including pharmacologic prophylaxis with anticoagulation, if appropriate. The anticipated enrollment is approximately 15,000 patients. The primary outcome is the occurrence of HA-VTE. Secondary outcomes include initiation of anticoagulation, reasons for not initiating anticoagulation among patients for whom it was recommended, and adverse bleeding events. Subgroup analyses will be conducted among patients with elevated HA-VTE risk.

**Discussion:**

This ongoing pragmatic randomized trial will provide a prospective assessment of a pediatric risk-prediction tool used to identify hospitalized patients at elevated risk of developing HA-VTE.

**Trial registration:**

ClinicalTrials.gov NCT04574895. Registered on September 28, 2020. Date of first patient enrollment: November 2, 2020.

## Background

Hospital-associated venous thromboembolism (HA-VTE) is an increasing cause of morbidity and mortality among pediatric patients, with increasing annual rates reported since 2001 [1, 2]. Children who develop HA-VTE experience longer hospitalizations and increased healthcare costs compared to their peers [3] and are at risk for developing lifelong medical complications [4, 5].

Risk-prediction models have been shown to identify patients at elevated risk for venous thromboembolism (VTE) better than physician judgment alone [6, 7]. However, these models are often limited to specific subpopulations—including patients undergoing surgery [8, 9], patients admitted to the intensive care unit [10] or to the general wards [11], and patients with malignancy [12]—and are mostly derived from case–control studies [13–16]. With the desire to create a single, general pediatric risk-prediction model applicable hospital-wide, we recently developed and temporally validated a general pediatric HA-VTE risk-prediction model using data available on admission from a large, single-center cohort [17].

We designed the Children’s Likelihood Of Thrombosis (CLOT) trial to evaluate whether the use of the risk-prediction model to guide the clinical care and management of pediatric patients at risk for HA-VTE lowers the rates of VTE and improves patient outcomes. Patients with an elevated probability of HA-VTE randomized 1:1 to the intervention arm have an additional review of their clinical course by a team of dedicated hematologists, who make recommendations to the admitting team regarding initiation of pharmacologic prophylaxis with anticoagulation, if appropriate. The primary aim is to determine whether HA-VTE rates are decreased in the intervention group, and secondary aims include evaluating whether patients are started on prophylactic anticoagulation and monitoring for bleeding events potentially associated with prophylaxis. We hypothesize that the use of the risk-prediction model to identify patients at elevated risk for HA-VTE will facilitate the timely and appropriate initiation of thromboprophylaxis and will decrease the rates of HA-VTE at our institution.

## Methods

### Design

The CLOT trial is a prospective, unblinded, pragmatic randomized trial evaluating the superiority of model-guided initiation of prophylactic anticoagulation versus usual care. Patient enrollment started on November 2, 2020, at Monroe Carell Jr. Children’s Hospital at Vanderbilt (MCJCHV) at Vanderbilt University Medical Center in Nashville, TN, USA. The trial was approved by the Vanderbilt University Medical Center Human Research Protections Program with a waiver of informed consent (IRB#201,629). The trial was registered with ClinicalTrials.gov prior to initiation of patient enrollment (ClinicalTrials.gov identifier: NCT04574895; date of trial registration: September 28, 2020; date of first patient enrollment: November 2, 2020). An independent safety monitoring committee is monitoring patient safety during the trial. The trial is investigator-initiated without additional funding; support for logistics and implementation is provided by the Vanderbilt Institute for Clinical and Translational Research and the Advanced Vanderbilt Artificial Intelligence Laboratory. The SPIRIT reporting guidelines and checklist were used when writing the trial protocol [18].

### Study sites and period

The CLOT trial is being conducted throughout the children’s hospital at MCJCHV and includes the general pediatrics wards, the pediatric critical care units (the pediatric intensive care unit and the pediatric cardiology intensive care unit), and the neonatal intensive care units. The hospital contains 343 beds, with 42 beds in the pediatric critical care units and 106 beds in the neonatal intensive care units. Enrollment began for all units simultaneously on November 2, 2020.

### Population

All patients 21 years of age and younger admitted to a pediatric unit at MCJCHV during the study period are enrolled in the study at the time an inpatient admission order is placed. Enrolled patients who are discharged from the hospital are eligible again if they are re-admitted during the study period. Patients admitted under observation status are not included.

### Consent

There have not been large, randomized trials to determine how to best identify pediatric patients at risk for developing VTE. Given the minimal risk to patients by calculating their risk of HA-VTE from a validated risk-prediction model, and the impracticality of consenting each patient admitted to MCJCHV prior to their risk being calculated, a waiver of informed consent was granted by the Vanderbilt University Medical Center Human Research Protections Program under 45 CFR 46.116. Prophylactic pharmacologic anticoagulation is commonly used for pediatric patients determined to be at elevated risk for developing thromboses to prevent future VTE development [19, 20]. This strategy has been shown in several studies to be safe for pediatric patients [20, 21]. All patients receive at least the current standard of care for initiation of prophylactic anticoagulation.

### Randomization and allocation

**Fig. 1:**
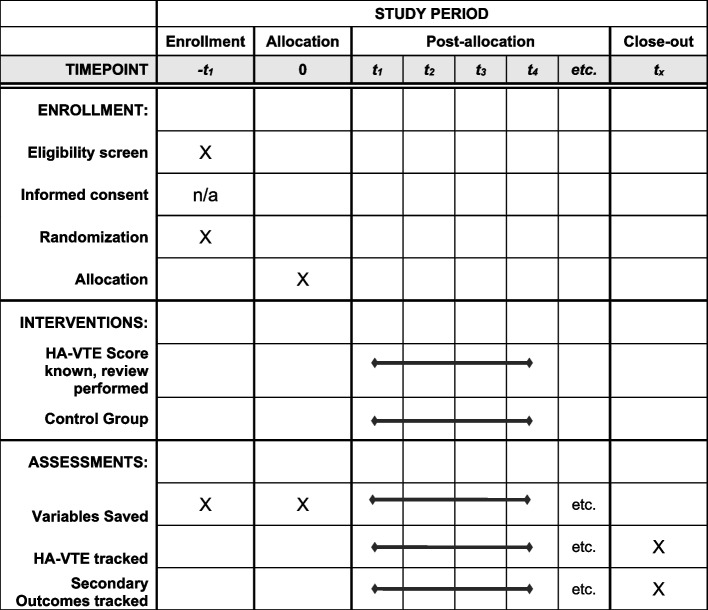
Schedule of enrollment, interventions, and assessments

Upon inpatient admission orders being placed for a patient to be admitted to MCJCHV, eligible patients are randomized by an Epic Best Practice Advisory that runs in the background and is not seen by providers. The advisory programmatically generates a random number to determine whether the patient is assigned to the control arm or intervention arm. The arm to which each patient is assigned is coded and saved for that admission encounter and then extracted into a daily report. See Fig. 1 for additional information.

### Concealment and blinding

The study principal investigator and the pediatric hematologists who review patients with elevated risk are unblinded to the intervention. Intervention assignment is masked to all other study personnel. Randomized patients are separated into two groups, concealed as “group A” and “group B.” The group assignments will remain concealed from the biostatistics team and safety monitoring committee until data analysis is complete. The principal investigator is not responsible for diagnosing HA-VTE.

### Study interventions

All pediatric patients admitted to MCJCHV during the study period have their predicted probability of HA-VTE calculated on admission and daily thereafter. Details on the calculation (i.e., the variables included in the risk-prediction model and their coefficients) can be found in our prior publication [17]. Patients in the intervention arm have their predicted probability reviewed by dedicated pediatric hematologists daily on weekdays. For those patients determined to be at elevated risk for development of HA-VTE, the hematologist performs a brief chart review to determine whether the patient might benefit from pharmacologic prophylaxis or whether they have known contraindications to anticoagulation (e.g., severe bleeding disorders, clinically significant hemorrhage, severe renal dysfunction, or anything in the opinion of the investigator that would jeopardize the safety of the patient). Generally, we define elevated risk as a predicted probability of HA-VTE ≥ 0.025. This cutpoint was determined from the distribution of predicted risk among an earlier validation cohort [17]. It was felt that this cutpoint would capture most patients who would develop a VTE while excluding most patients who would not. Therefore, the intervention would be targeted toward the patients at higher risk. The hematologist provides recommendations to the primary admitting team, including initiation of prophylactic pharmacologic anticoagulation. Whether or not the recommendation was accepted, and the reason for not accepting the recommendation, is captured for future analysis. All patients in the study will continue to receive the current standard-of-care treatment.

### Data collection

For this pragmatic trial, we are using data collected in routine clinical care and electronically extracted from our institution’s electronic health record (EHR). Data are secured confidentially in an institutional patient data management system and on secured servers. Data collected include demographic, laboratory, and clinical data. Data are extracted daily from an analytical report using Epic Clarity and are presented in an organized fashion, including all of the risk factors included in the risk-prediction model: patient age; history of thrombosis; cardiology or infectious diseases consult, or were admitted to that clinical team; cancer diagnosis prior to or during the encounter; surgical procedures during the encounter; selected laboratory information; and presence of a central venous catheter before or during the encounter [17]. The information presented in the report is saved automatically to secure servers and is separated into intervention and control groups. Patients in the intervention arm who have undergone hematology review have additional data collected such as the date their clinical review occurred, the predicted probability of HA-VTE at the time of intervention, and the outcome of the recommendation. Two random patients in the control arm are audited weekly to ensure enrollment criteria are being met. Patients who developed HA-VTE are identified through an internal review based on radiology reports, and information such as the date, type of HA-VTE, and study arm are collected. Adverse event data are collected in an ongoing fashion. All data are saved in an institutional patient data management system (REDCap). Access to the final trial dataset will be limited to the primary study investigators and biostatisticians.

### Primary outcome

The primary outcome is the diagnosis of VTE during hospitalization, with data collected as described above. To ensure all cases of HA-VTE are captured, a separate dataset will be obtained after study closure from the EHR using ICD-9/10 codes for acute VTE. A subset of these will be reviewed for accuracy, as done in the prior model development work [17]. The primary endpoint measure will be calculated as the proportion of encounters during which the patient experienced a HA-VTE event compared between the intervention and control arms.

### Secondary outcomes

Secondary outcomes include additional clinical outcomes and possible adverse events. Clinical outcomes include the total number of all patients who initiate prophylactic anticoagulation, by study arm, and the total number of patients at elevated risk for HA-VTE who are begun on prophylactic anticoagulation, by study arm. Further evaluation will also include the total number of patients who initiate anticoagulation medications compared to the total number of patients for which initiation of anticoagulation was recommended by the research team. To ensure patient safety, we will also assess the frequency of bleeding-related adverse events per number of patients begun on prophylactic anticoagulation, scored using the modified WHO bleeding scale, by study arm, during hospitalization.

### Power calculation

We anticipate an enrollment of at least 15,000 patients that will be randomized 1:1 to the intervention and control groups. With at least 7500 patients per group, a two-sample Fisher’s exact test achieves at least 80% power to detect an absolute risk reduction from 1.0% in the control group (the historical rate of HA-VTE at our institution [17]) to 0.595% in the intervention group.

### Safety monitoring committee

A safety monitoring committee (SMC) was constituted prior to study initiation. The independent SMC consists of three pediatric physicians, including a general pediatrician and pediatric intensivist from the study institution with experience in risk-prediction studies and pediatric thromboses, respectively, and a pediatric hematologist from another institution. The SMC reviews the study for safety every 6 months and evaluates whether the study should continue as is, continue with modifications, or be terminated and unblinded due to safety concerns. There are no pre-determined stopping rules. The SMC is also available to convene as needed to evaluate adverse events and serious adverse events during the study period.

### Statistical analysis principles

All analyses will be performed using reproducible research methods in the current version of R (R Foundation for Statistical Computing, Vienna, Austria). Primary analyses will be conducted at the encounter level, while secondary analyses will be conducted at the patient level (patients can potentially experience multiple encounters during the study period). Primary analyses will be conducted by intention-to-treat; secondary analyses will be as treated, considering whether treatment recommendations were followed by the admitting team. Continuous variables will be summarized using median and interquartile range or mean and standard deviation, depending on their distribution. Categorical variables will be summarized using frequencies and proportions. The rate of VTE events (primary outcome), the proportion of patients receiving prophylactic anticoagulation (secondary outcome), and the rate of bleeding-related adverse events (secondary outcome) will be compared between the intervention and control arms using unadjusted risk differences with 95% confidence intervals. We have not specified a priori any adjustment variables to include in multivariable models. Any variable with a clinically meaningful imbalance between the intervention and control groups will be adjusted for in multivariable regression models; given the large sample size, we do not anticipate any such imbalances. For the primary outcome, a two-sided *p*-value (obtained from Pearson’s chi-square test) of < 0.05 will indicate statistical significance. We will avoid formal statistical hypothesis testing for secondary outcomes and subgroup analyses.

### Analytic rationale

Rates of pediatric VTE have been reported to be increasing nationally and internally at MCJCHV. This study evaluates the use of a novel risk-prediction tool utilized in conjunction with a targeted intervention, which is a personalized pediatric hematology review. The primary and secondary analyses will evaluate the effect of the intervention across a spectrum of predicted VTE risk and in a general pediatric patient population.

### Primary analysis

The primary analysis will be an intention-to-treat comparison of HA-VTE rates by study arm in the overall patient population using an uncorrected chi-square test.

### Secondary analysis

Secondary subgroup analyses will be performed by age, gender, risk of HA-VTE, primary diagnoses, surgical vs. non-surgical, and consultants involved. Secondary as-treated analyses will be performed among the strata of high-risk patients (predicted probability ≥ 0.025) randomized to usual care, to the intervention but treatment recommendations were not followed, and to the intervention and treatment recommendations were followed.

### Missing data

Any missing data in variables used to generate the predicted probability of HA-VTE are replaced with the median value among the cohort of patients used to derive the model [17]. Missing data will not arise for outcomes because the occurrence of HA-VTE and bleeding events during hospitalization are captured by diagnosis codes in the EHR. Initiation of anticoagulation medications during hospitalization is fully captured by order information within the EHR.

### Presentation of results

We anticipate submitting the results of the study to a peer-reviewed journal upon completion of the trial. We will also present the data at relevant national and international conferences.

### Protocol amendments

In the event of important protocol modifications, changes will be communicated to the SMC, IRB, and the clinicaltrials.gov registry will be updated accordingly.

## Discussion

The CLOT trial is a large pragmatic randomized trial currently being conducted to assess whether the use of a novel VTE risk-prediction model to identify pediatric patients who are at elevated risk for developing HA-VTEs for targeted review by a pediatric hematologist improves overall rates of HA-VTEs.

Prior to study initiation, the study team met with pediatric providers across multiple subspecialties at MCJCHV to design a study that would best meet the needs of the pediatricians. Prior studies and similar adult studies conducted at our institution involved Epic Best Practice Advisories that were seen and acted on by the primary admitting team. Pediatricians, however, were concerned that with this style of study, the study team’s recommendations would not be followed without first obtaining input from the pediatric hematology team. To minimize additional work on behalf of the pediatric hematology clinical service, we elected to design the study using a study team pediatric hematologist to review patients found to be at elevated risk and then approaching the admitting teams directly.

We believe that the final trial design works well within the pediatric hospital setting and allows for recommendations and conversations to be had between the hematology research team and then admitting pediatric teams. The study design is adaptable to future scientific endeavors.

## Trial status

The CLOT trial is an ongoing, pragmatic randomized trial to compare the use of a novel risk-prediction model to identify pediatric hospitalized patients at elevated risk of developing an HA-VTE to the current standard of care. Patient enrollment began on November 2, 2020, and is scheduled to continue until at least 15,000 hospital admissions are captured. Although the trial was anticipated to last 1 year, the ongoing COVID-19 pandemic impacted the patient volume at MCJCHV; the trial will be conducted until the target sample size is accrued.

## Data Availability

The datasets generated during and/or analyzed during the current study are not publicly available due to them containing information that could compromise participant privacy but are available from the corresponding author on reasonable request.

## References

[1] Raffini L, Huang YS, Witmer C, Feudtner C (2009). Dramatic increase in venous thromboembolism in children’s hospitals in the United States from 2001 to 2007. Pediatrics.

[2] Mahajerin A, Croteau SE (2017). Epidemiology and risk assessment of pediatric venous thromboembolism. Front Pediatr.

[3] Goudie A, Dynan L, Brady PW, Fieldston E, Brilli RJ, Walsh KE (2015). Costs of venous thromboembolism, catheter-associated urinary tract infection, and pressure ulcer. Pediatrics.

[4] Kumar R, Rodriguez V, Matsumoto JM, Khan SP, Weaver AL, McBane RD (2015). Prevalence and risk factors for post thrombotic syndrome after deep vein thrombosis in children: a cohort study. Thromb Res.

[5] Goldenberg NA (2005). Long-term outcomes of venous thrombosis in children. Curr Opin Hematol.

[6] Ellis HB, Sabatino MJ, Clarke Z, Dennis G, Fletcher AL, Wyatt CW (2019). The importance of a standardized screening tool to identify thromboembolic risk factors in pediatric lower extremity arthroscopy patients. J Am Acad Orthop Surg.

[7] Cunningham AJ, Dewey E, Lin S, Haley KM, Burns EC, Connelly CR (2020). Pediatric trauma venous thromboembolism prediction algorithm outperforms current anticoagulation prophylaxis guidelines: a pilot study. Pediatr Surg Int.

[8] Sherrod BA, McClugage SG, Mortellaro VE, Aban IB, Rocque BG (2019). Venous thromboembolism following inpatient pediatric surgery: analysis of 153,220 patients. J Pediatr Surg.

[9] Connelly CR, Laird A, Barton JS, Fischer PE, Krishnaswami S, Schreiber MA (2016). A clinical tool for the prediction of venous thromboembolism in pediatric trauma patients. JAMA Surg.

[10] Arlikar SJ, Atchison CM, Amankwah EK, Ayala IA, Barrett LA, Branchford BR (2015). Development of a new risk score for hospital-associated venous thromboembolism in critically-ill children not undergoing cardiothoracic surgery. Thromb Res.

[11] Atchison CM, Arlikar S, Amankwah E, Ayala I, Barrett L, Branchford BR (2014). Development of a new risk score for hospital-associated venous thromboembolism in noncritically ill children: findings from a large single-institutional case-control study. J Pediatr.

[12] Mitchell L, Lambers M, Flege S, Kenet G, Li-Thiao-Te V, Holzhauer S (2010). Validation of a predictive model for identifying an increased risk for thromboembolism in children with acute lymphoblastic leukemia: results of a multicenter cohort study. Blood.

[13] Sharathkumar AA, Mahajerin A, Heidt L, Doerfer K, Heiny M, Vik T (2012). Risk-prediction tool for identifying hospitalized children with a predisposition for development of venous thromboembolism: Peds-Clot clinical Decision Rule. J Thromb Haemost.

[14] Kerlin BA, Stephens JA, Hogan MJ, Smoyer WE, O'Brien SH (2015). Development of a pediatric-specific clinical probability tool for diagnosis of venous thromboembolism: a feasibility study. Pediatr Res.

[15] Jaffray J, Branchford B, Goldenberg N, Malvar J, Croteau SE, Silvey M, et al. Development of a risk model for pediatric hospital-acquired thrombosis: a report from the children’s hospital-acquired thrombosis consortium. J Pediatr. 2021;228:252–9.e1 10.1016/j.jpeds.2020.09.016 PMC7752847 32920105

[16] Goel R, Dhillon J, Malli C, Sahota K, Seehra P, Streiff MB (2013). A risk-prediction model for identifying venous thromboembolism in hospitalized pediatric patients: a single institution retrospective case-control analysis. Blood.

[17] Walker SC, Creech CB, Domenico HJ, French B, Byrne DW, Wheeler AP (2021). A real-time risk-prediction model for pediatric venous thromboembolic events. Pediatrics.

[18] Chan A-W, Tetzlaff JM, Gøtzsche PC, Altman DG, Mann H, Berlin J, Dickersin K, Hróbjartsson A, Schulz KF, Parulekar WR, Krleža-Jerić K, Laupacis A, Moher D (2013). SPIRIT 2013 explanation and elaboration: guidance for protocols of clinical trials. BMJ.

[19] Faustino EV, Hanson S, Spinella PC, Tucci M, O'Brien SH, Nunez AR (2014). A multinational study of thromboprophylaxis practice in critically ill children. Crit Care Med.

[20] Raffini L, Trimarchi T, Beliveau J, Davis D (2011). Thromboprophylaxis in a pediatric hospital: a patient-safety and quality-improvement initiative. Pediatrics.

[21] Stem J, Christensen A, Davis D, Raffini L (2013). Safety of prophylactic anticoagulation at a pediatric hospital. J Pediatr Hematol Oncol.

